# Contrast Agent‐Free 3D Renal Ultrafast Doppler Imaging Reveals Vascular Dysfunction in Acute and Diabetic Kidney Diseases

**DOI:** 10.1002/advs.202303966

**Published:** 2023-10-17

**Authors:** Donghyeon Oh, Donghyun Lee, Jinseok Heo, Jooyoung Kweon, Uijung Yong, Jinah Jang, Yong Joo Ahn, Chulhong Kim

**Affiliations:** ^1^ Departments of Electrical Engineering Convergence IT Engineering Medical Science and Engineering Mechanical Engineering and Medical Device Innovation Center Pohang University of Science and Technology (POSTECH) Cheongam‐ro 77, Nam‐gu Pohang Gyeongbuk 37673 Republic of Korea

**Keywords:** 3D ultrafast doppler, acute kidney injury, diabetic kidney disease, renal vascular imaging

## Abstract

To combat the irreversible decline in renal function associated with kidney disease, it is essential to establish non‐invasive biomarkers for assessing renal microcirculation. However, the limited resolution and/or vascular sensitivity of existing diagnostic imaging techniques hinders the visualization of complex cortical vessels. Here, a 3D renal ultrafast Doppler (UFD) imaging system that uses a high ultrasound frequency (18 MHz) and ultrahigh frame rate (1 KHz per slice) to scan the entire volume of a rat's kidney in vivo is demonstrated. The system, which can visualize the full 3D renal vascular branching pyramid at a resolution of 167 µm without any contrast agent, is used to chronically and noninvasively monitor kidneys with acute kidney injury (AKI, 3 days) and diabetic kidney disease (DKD, 8 weeks). Multiparametric UFD analyses (e.g., vessel volume occupancy (VVO), fractional moving blood volume (FMBV), vessel number density (VND), and vessel tortuosity (VT)) describe rapid vascular rarefaction from AKI and long‐term vascular degeneration from DKD, while the renal pathogeneses are validated by in vitro blood serum testing and stained histopathology. This work demonstrates the potential of 3D renal UFD to offer valuable insights into assessing kidney perfusion levels for future research in diabetes and kidney transplantation.

## Graphical abstract

Contrast agent‐free 3D renal ultrafast Doppler (UFD) imaging system noninvasively visualizes the delicate vascular network of the kidney in vivo at fine resolution (167 µm) without contrast agent. The rat's kidneys with acute kidney injury (72 h) and diabetic kidney disease (8 weeks) are chronically monitored, and their hemodynamic and morphological changes are quantitatively followed up via multiparametric UFD analyses.

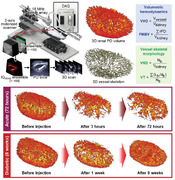

## 1 Introduction

Kidney disease, also called nephropathy, is a highly prevalent affliction that significantly impacts public health. Kidney disease affects over 1 in 7 adults in the United States, with nearly 1 in 3 individuals with diabetes and 1 in 5 individuals with high blood pressure being impacted by this condition.^[^ ^1^ ^]^ Kidney disease can be acute or chronic. Acute kidney injury (AKI) is a short‐term sudden loss of kidney function occurring in 13.3 million people, and yielding 1.7 million deaths per year.^[^ ^2^ ^]^ Diabetic kidney disease (DKD), a progressive decline in kidney filtration as a result of persistent diabetes mellitus, is the most common diabetic complication, developed in ≈40% of all diabetic patients.^[^ ^3^ ^]^ One of the most lethal aspects of kidney disease is a predominantly irreversible decline in renal function, necessitating life‐long kidney dialysis or donor kidney transplantation for survival.

A kidney is a highly perfused organ that is directly connected to the abdominal aorta and inferior vena cava to filter metabolic waste products from the bloodstream. It is well‐established that kidney disease is closely associated with cardiovascular diseases such as hypertension and diabetes. In clinical practice, kidney function loss is commonly evaluated by the glomerular filtration rate (GFR), which is inductively estimated from the blood serum creatinine (sCr) level.^[^ ^4^ ^]^ However, the sCr is not solely affected by the kidney's functional status, which often leads to misprediction of the GFR.^[^ ^5^ ^]^ Previous studies have used miniaturized flowmeters to directly measure renal arterial flow.^[^ ^6^, ^7^ ^]^ However, this method requires invasive surgical procedures and cannot represent local perfusion changes. Preclinical studies using microscopic imaging techniques to image the renal microvasculature have shed light on the nature of vascular rarefaction in AKI or DKD,^[^ ^8^, ^9^ ^]^ but microscopic techniques are restricted to *ex vivo* studies. To image vasculatures in depth in vivo, photoacoustic imaging (PAI), a hybrid optical and acoustical modality, can reach the deepest among optical imaging techniques.^[^ ^10^, ^11^, ^12^, ^13^, ^14^, ^15^, ^16^ ^]^ However, because of strong optical attenuation, the identification of vascular structures may be limited to the superficial level. To target deep renal vessels, noninvasive diagnostic imaging techniques, such as computed tomography (CT) angiography, and magnetic resonance imaging (MRI), have been used.^[^ ^17^, ^18^ ^]^ However, these imaging modalities have insufficient resolution to visualize intricate interlobular vessels, and they require exogenous contrast agents.

Taking advantage of technical advances in signal acquisition and processing, ultrafast Doppler imaging (UFD) attains very high ultrasound (US) sampling speeds, with frame rates of up to 20 KHz. The resulting ultrasound imaging (USI) possesses the vascular sensitivity needed in cardiovascular,^[^ ^19^, ^20^ ^]^ hepatic,^[^ ^21^ ^]^ nephrological,^[^ ^22^ ^]^ and neurological research.^[^ ^23^, ^24^, ^25^, ^26^ ^]^ The Doppler signal intensity itself represents the instantaneous blood volume, hence UFD can capture both the vascular morphology and local hemodynamics of the organ. With the use of a US contrast agent, super‐resolution US techniques, including ultrasound localization microscopy, have overcome the spatial resolution barrier to US imaging beyond the acoustic diffraction limit.^[^ ^27^, ^28^ ^]^ Nonetheless, previous UFD or super‐resolution US studies on the kidneys have been limited to 2D imaging of blood vessels,^[^ ^29^, ^30^, ^31^, ^32^ ^]^ which is insufficient to represent the 3D vasculatures of the entire kidney. Furthermore, super‐resolution techniques require continuous injection of a contrast agent throughout the lengthy data acquisition period and also require localization and trajectory tracking algorithms with a high computational cost. In contrast, UFD imaging does not require exogenous contrast agents and saves time and computational expenses, making it easier to scan the entire kidney volume in 3D.

Here, we demonstrate a contrast agent‐free 3D renal UFD imaging system that can visualize the full renal vascular branching pyramid. Driven by an 18‐MHz high‐resolution linear array US probe at an ultrafast frame rate (1000 Hz), the resultant power Doppler (PD) volume captures the whole renal vascular network of a rat's kidney with the finest diameter of 167 µm. We further monitor the kidneys with rhabdomyolysis AKI and type‐I DKD for 3 days and 8 weeks, respectively. During the pathologic progression, renal vascular features are quantitatively assessed by hemodynamic and vessel morphologic parameters, namely the vessel volume occupancy (VVO), fractional moving blood volume (FMBV), vessel number density (VND), and vessel tortuosity (VT). A notable difference is found between the parametric changes in the AKI and DKD models. In the AKI model, ≈35% decreases in VVO, FMBV, and VND are attributed to rapid vascular rarefaction. In comparison, in the DKD model, chronic vascular degeneration was notable: VVO, FMBV, and VND were ≈23% lower than in the control group, while tortuous morphological change was marked by an ≈9% increase in VT. The development and progression of kidney diseases were confirmed through in vitro blood serum tests and histopathology of the collected kidneys. Our research illustrates that 3D renal UFD has the potential to provide significant insights into the physiology of renal perfusion, which could have potential applications in studying diabetes and kidney transplantation.

## 2 Results

### 2.1 The 3D Renal UFD System and Its Data Acquisition and Image Processing Procedures

**Figure 1:**
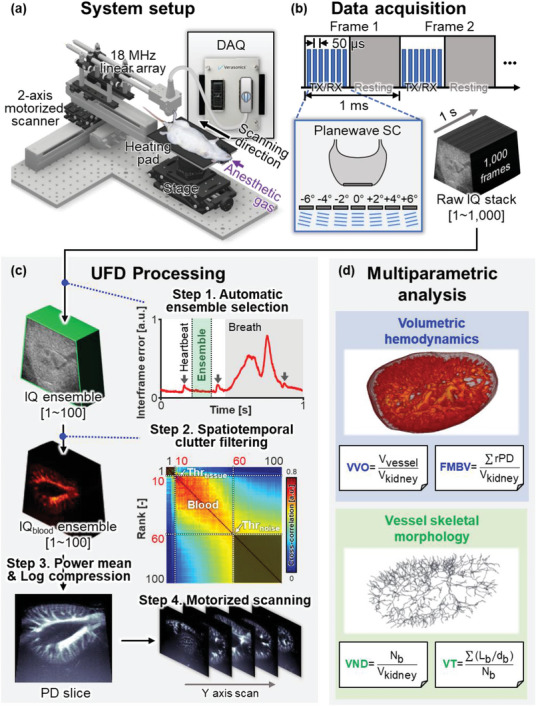
Data acquisition (DAQ), image processing, and multiparametric analyses in a 3D renal ultrafast Doppler (UFD) system. a) Configuration of the 3D renal UFD system. b) Data acquisition scheme. c) Flowchart of off‐line UFD postprocessing. d) Multiparametric UFD analyses: vessel volume occupancy (VVO); fractional moving blood volume (FMBV); vessel number density (VND); and vessel tortuosity (VT). SC, spatial compounding; IQ, in‐phase quadrture data; IQ_blood_, blood in‐phase quadrature data; a.u., arbitrary unit; Thr_tissue_, tissue signal threshold; Thr_noise_, noise signal threshold; PD, power Doppler; *V*_vessel_, vessel mask volume; *V*_kidney_, kidney mask volume; rPD, relative power Doppler; *N*_b_, total number of vessel branches; *L*_b_, actual vessel branch length; and *d*_b_, linear distance of vessel branch.

**Figure** **1**a shows the 3D renal UFD system schematically, and further system details are in Section 4.1. The US data were acquired using an 18 MHz linear array US probe (L22‐14vX, Vermon, France) that was coupled with a Vantage 256 data acquisition system (DAQ) (Verasonics, WA, USA) (Figure 1a). The US probe was positioned to view the transverse plane of the kidney and adjusted to fully include the kidney in the imaging field‐of‐view (FOV). Each US frame was acquired with 20 KHz 7‐angle compounded planewave mode, yielding a B‐mode frame rate of 1 KHz (Figure 1b). The acquired raw radiofrequency (RF) frames were instantly reconstructed into images, resulting 1000 in‐phase/quadrature (IQ) images. The entire kidney volume was scanned by sliding the linear array US probe directly on the rat's gel‐coated skin with a motorized stage.

The acquired IQ frames were subsequently processed offline into PD images (Figure 1c). First, a 100‐frame ensemble was selected from the original 1000 IQ frames per elevational slice, using an automatic ensemble selection algorithm. To mitigate respiratory or major cardiac motion artifacts, the criterion for ensemble selection was the least interframe error difference. Consequently, consecutive frames during a systolic phase without breathing were automatically collected (Figure S1 and Movie S1, Supporting Information). Second, the blood IQ signals were filtered from tissue signals with a spatiotemporal clutter filter based on singular value decomposition (SVD). This filter exhibits higher sensitivity and is more robust to tissue motion than a high‐pass filter‐based temporal clutter filter.^[^ ^22^ ^]^ The filtering yielded IQ data containing only the blood signal and random noise. Finally, PD images were composed by averaging the power intensity of the blood IQ signal ensemble. The overall procedure was repeated along the *y*‐axis, ultimately capturing the 3D PD volume of the entire kidney (Figure S2 and Movie S2, Supporting Information). The detailed postprocessing procedure is described in Section 4.2.

To quantitatively evaluate the hemodynamics and morphology of the acquired kidney PD volume, we performed multiparametric analyses using four selected parameters: VVO, FMBV, VND, and VT (Figure 1d). The selected parameters are subcategorized according to their key features: VVO and FMBV are volumetric hemodynamic parameters derived from the PD volumes, and the VND and VT are quantified from a skeletonized branch structure demonstrating the vascular morphology. VVO reflects the ratio of the vessel volume to the kidney volume. FMBV is an average of a relative PD intensity regarding 100% at the major vessel, and it indicates the perfusion rate of the blood. VND is calculated by dividing the total number of skeletal branches by the kidney volume, and the VT is estimated by a distance metric, the ratio between the actual and shortest path length of the branch. The statistical validation confirms the consistency of intersubject and intrasubject repeatability in the UFD analysis, utilizing data from a stable cohort of experimental subjects in a preinjection state (*n* = 16) as well as from time‐series imaging sessions of a healthy subject (*n* = 3) (Figure S10, Supporting Information). The quantification procedure is described in detail in Section 4.5.

### 2.2 3D Structural UFD Images of Renal Vascular Pyramid In Vivo

**Figure 2:**
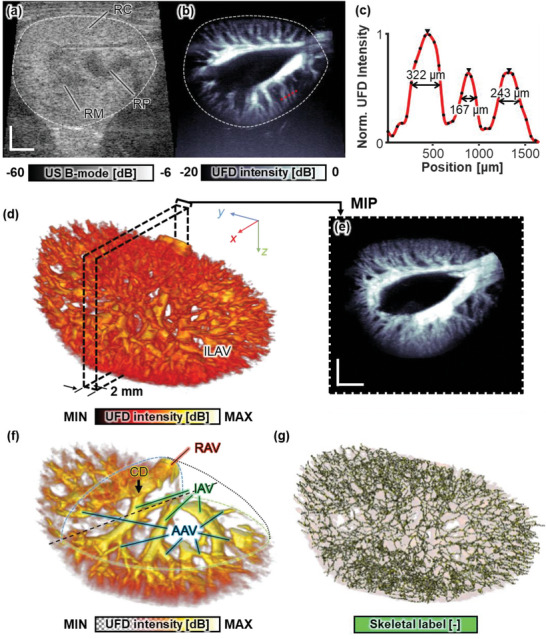
3D renal UFD visualizations of rat kidney vascular structures in vivo. A central transverse view of a kidney is displayed in a) an ultrasound (US) B‐mode image and b) a UFD image (Movie S2, Supporting Information). c) A 1D vessel profile of 3 interlobular vessels cut along the red dotted line in (b). Vessel diameters were estimated from the measured full width at half‐maximum values of the peak widths. d) 3D rendering of a whole renal vascular network, showing rich ILAV vasculature in the renal cortex (Movie S3, Supporting Information). e) A maximum intensity projection (MIP) view of the central ±1 mm block defined by the black dotted box in (d). f) A quadrant sectional view with adjusted transparency showing the branching vessel hierarchy. g) Vessel skeleton extracted from the PD volume (Movie S4, Supporting Information). RC, renal cortex; RM, renal medulla; RP, renal pelvis; ILAV, interlobular artery‐vein; RAV, renal artery‐vein; CD, caudal division; IAV, interlobar artery‐vein; and AAV, arcuate artery‐vein. Scale bar = 2 mm.

**Figure** **2** shows the 3D structural renal vascular network of a healthy rat in vivo. The upper margin of the kidney is right below the skin layer, ≈3 mm distant from the probe surface in the B‐mode image (Figure 2a). The renal cortex, medulla, and pelvis are distinguishable with difficulty, mainly by their distances from the renal capsule and their distinctive echogenicity. Further, only a few major blood vessels are observable, such as the renal artery and vein (RAV) in the valley of the hilum or the interlobar artery and veins (IAV) running down the corticomedullary junction. In marked contrast, a corresponding PD image of the same location reveals finer vascular features, such as the arcuate arteries and veins (AAV) and the interlobular arteries and veins (ILAV) dividing from the IAV (Figure 2b). The AAV and ILAV branch orthogonally from the corticomedullary junction toward the kidney capsule. Additionally, the visible vasculature in the PD image helps to distinguish the renal boundary from the surrounding adipose tissue (Figure 2b), whereas, in the B‐mode image, the vasculature appears isoechoic to the kidney tissue (Figure 2a). We estimated the vessel diameters by analyzing three neighboring ILAV in the dorsal hemisphere (Figure 2c). The full‐width at half‐maximum of each peak from the line profile cut along the red dotted line in Figure 2b confirms that the neighboring vessel diameters from left to right are 322, 167, and 243 µm, respectively. We regard the vessel diameter of the thinnest middle vessel as the image resolution value.

To visualize the entire kidney's vascular connectivity, we assembled sequential PD slices into the whole 3D PD volume, extending longitudinally from the cranial to the caudal end of the kidney (Figure 2d; Movie S3, Supporting Information). In the maximum intensity projection image of a central ±1 mm range, clear vascular connectivity is well visualized from the hilum to the distal capsule (Figure 2e). Figure 2f shows the inner vascular network branching hierarchy from the RAV to the ILAV. A pair of dorsal and ventral renal arteries and one renal vein separate at a hilar junction into two major divisions referred to as the cranial and caudal divisions. At each division point, the RAVs are divided into four IAV strands that extend towards the periphery of the kidney. Next, the AAV smoothly forms an arc parallel to the kidney surface. Finally, the AAV branches into the ILAV toward the kidney capsule, and these branches are the thinnest identifiable vascular hierarchy in the 3D UFD volume image. In addition to showing fine connectivity, the 3D vessel skeleton also delineates the whole renal vascular pyramid well (Figure 2g; Movie S4, Supporting Information). RBCs serve as acoustic scatterers in the US image, and this scattering phenomenon becomes a key principle to visualize blood vessels from the UFD images. RBCs are filtrated by glomeruli and do not permeate through renal tubules, which makes the renal tubules unidentifiable from the UFD images.

### 2.3 Serial Monitoring of Renal Vascular Change In Vivo in an AKI Rat Model

**Figure 3:**
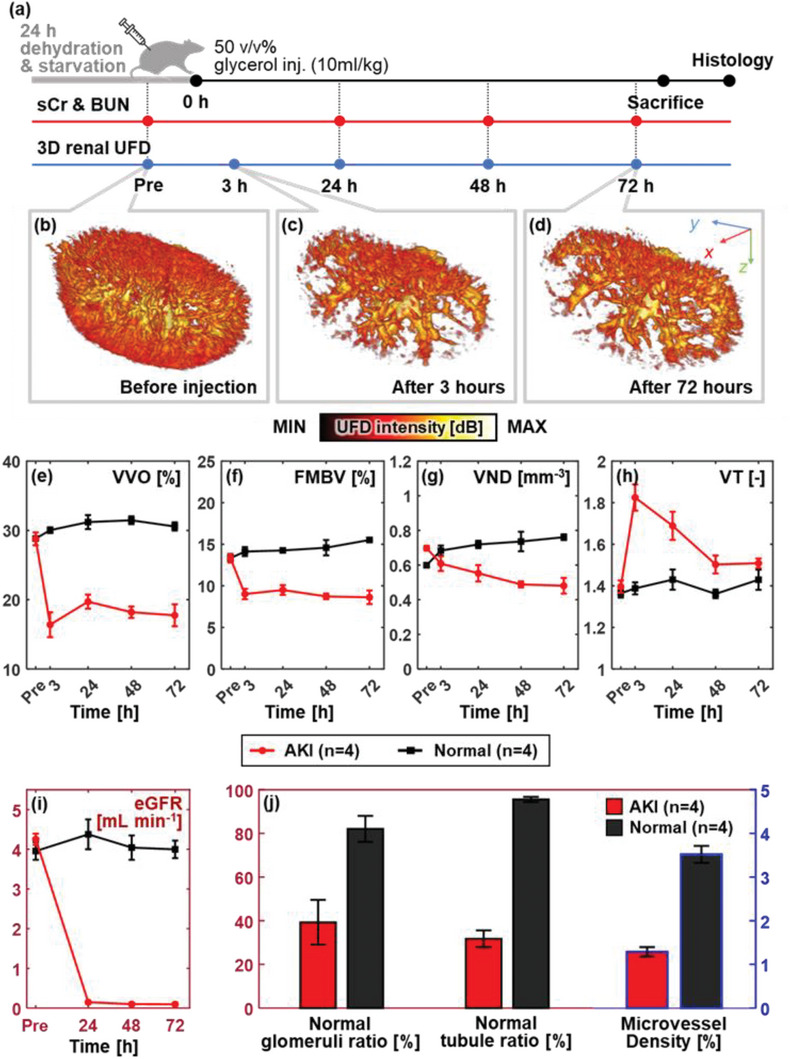
Progressive vascular rarefaction of rat kidneys in a glycerol‐induced AKI model. a) Experimental protocol. Representative 3D renal UFD volume at b) preinjection, c) 3‐h, and d) 72‐h postinjection of 50 v/v% glycerol (Movie S5, Supporting Information). Changes in UFD vascular parameters: e) vessel volume occupancy (VVO), f) fractional moving blood volume (FMBV), g) vessel number density (VND), and h) vessel tortuosity (VT) over time. i) The decrease in the estimated glomerular filtration rate (eGFR), calculated from serum creatinine (sCr), blood urea nitrogen (BUN), and body weight, signifies renal dysfunction. j) Quantitative histopathology results given as normal glomeruli ratio, normal tubule ratio, and microvessel density remarks renal microcirculatory disorder from AKI group (Figure S5, Supporting Information). Data are plotted as mean ± standard errors.

To demonstrate the capability of detecting vascular change under renal pathogenesis, we monitored a glycerol‐induced AKI rat model for 72 h. The entire experimental timeline is displayed in **Figure** **3**a. Glycerol‐saline solution was administered intramuscularly to induce rhabdomyolysis in four Sprague–Dawley (SD) rats, while an equal volume of saline was injected into a control SD rat group (*n* = 4). Glycerol induces excessive dehydration and breaks down muscle tissue rapidly, and the consequent immoderate release of myoglobin inflicts critical damage on the kidney by inducing oxidative stress and inflammatory responses.^[^ ^33^, ^34^ ^]^ The rats were chronically imaged preinjection, then at 3, 24, 48, and 72 h after injection. Concentrations of sCr and blood urea nitrogen (BUN), the waste products filtered out during renal circulation, were monitored in vitro during the in vivo imaging experiments. In the end, the rats were sacrificed, and their collected kidneys were prepared and mounted as histological slides to confirm AKI‐related lesions.

During the imaging experiments, we noticed a broadening of the hypoechoic area in the B‐mode image and a significant decrease of entire vascularity by means of number and contrast in the PD image. Most of the ILAVs vanished after injection, revealing the inner RAV, IAV, and AAV structures. Compared to the preinjection state (Figure 3b), the most extreme vascular rarefaction was observed at 3‐h postinjection (Figure 3c). The vascular volume has partially recovered by 24‐h postinjection, however, the vessel continues to regress to the final 72 h timepoint (Figure 3d; Figure S3 and Movie S5, Supporting Information). The rapid vascular rarefaction of the AKI kidneys is also well demonstrated by the changes in the UFD parameters over time, while no significant change is seen in the control group (Figure 3e–h). Overall, the VVO (Figure 3e), FMBV (Figure 3f), and VND (Figure 3g) of the AKI group show steep decreases of ≈35% at 72‐h postinjection, compared to the values of the preinjection state. Notably, the VVO has rapidly declined by 3 h, has recovered somewhat by 24 h, but then continuously decreases to 72 h. The FMBV shows a similar trend to that of the VVO, but with less variation in scale. The VND consistently decreases, with fairly quick degradation through 48 h, then reaches the lowest value at 72‐h postinjection. Contrary to the declining trend, the VT (Figure 3h) first increases by ≈31%, reaching its maximum value at 3 h, then gently decreases thereafter. The VT values for the AKI and normal groups differ largely at the 3‐h timepoint, but the difference becomes smaller by the 24‐, 48‐ and 72‐h timepoints, i.e., the early large changes in VT are temporary. This finding reflects the significant loss of ILAVs in the early phase, which are relatively straighter than the IAV and the AAV (Figure S3, Supporting Information). Such findings could be related to the etiology of rhabdomyolysis in AKI, which excessively discharges myoglobin, obstructs tubules, and ultimately leads to decreased glomerular filtration.^[^ ^35^ ^]^ Overall, our in vivo imaging results show a significant decrease in renal vascular hemodynamics, which correlates with the reduced renal blood flow (RBF) in the rhabdomyolysis AKI model.^[^ ^34^ ^]^

The imaging results were validated via in vitro serum assay (Figure 3i) and histopathological images (Figure 3j–l). The high sCr and BUN concentrations indicate functional filtration loss in the kidney. In the AKI model, the sCr and BUN had increased by 15.3‐ and 8.3‐fold, respectively, by 24‐h postinjection, and continuously increased further, reaching maxima of 22.5‐ and 14.1‐fold, respectively (Figure S4, Supporting Information). In comparison, the BUN and sCr values from the normal group remained stable throughout 3 days to their original level. Further, the estimated glomerular filtration rate (eGFR) was calculated using a large cohort‐based eGFR equation,^[^ ^36^ ^]^ considering the in vitro results and the measured body weight as input parameters. As seen in Figure 3i, the eGFR value in the AKI group (0.10 ± 0.03 mL min^−1^), is 41.7 times lower than that of the normal group (4.00 ± 0.22 mL min^−1^). From the quantitative histologic analysis, the AKI group shows 52.3%, 66.8%, and 62.2% decreases in the normal glomeruli ratio, normal tubule ratio, and microvessel density, respectively, to those of the normal kidneys (Figure 3j). In specific, severe fibrosis is identified from the glomeruli and blood vessels, together with major tubular necrosis (Figure S5, Supporting Information). In summary, our in vitro results well demonstrate the vascular flow decline identified from the in vivo UFD volume and the kidney dysfunction is well validated.

### 2.4 Chronical Monitoring of Renal Vascular Change In Vivo in a DKD Rat Model

**Figure 4:**
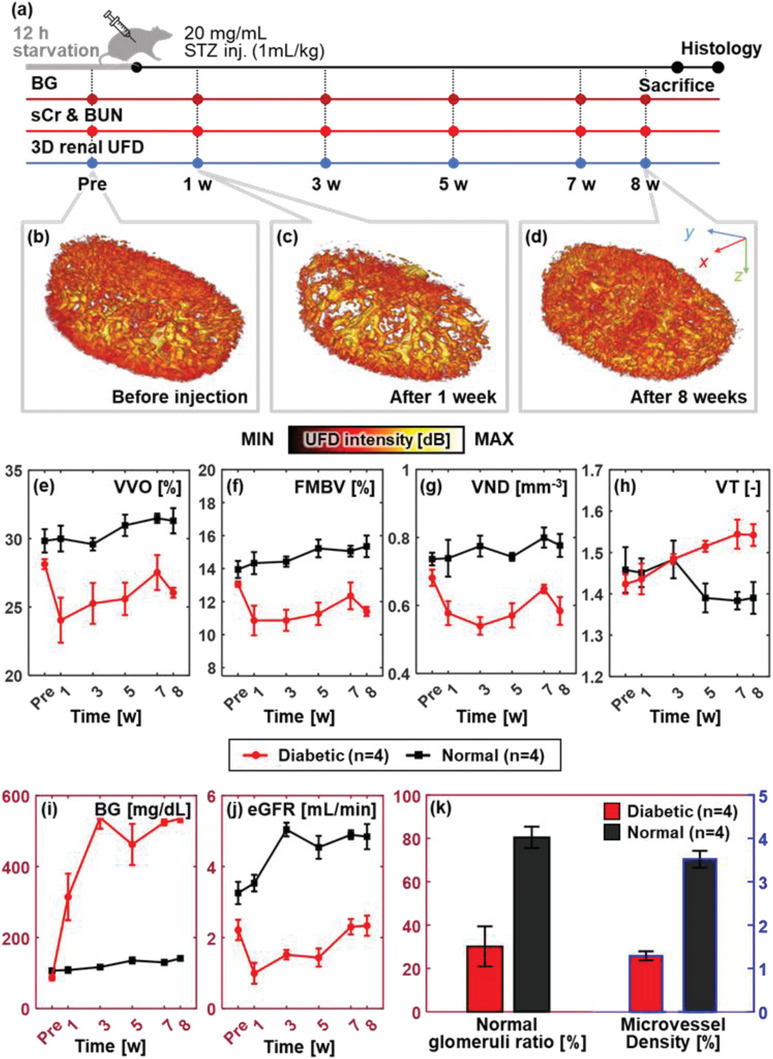
Long‐term vascular decline of rat kidneys in a streptozotocin‐induced diabetes model. a) Scheme of the experimental protocol. Representative 3D UFD volumes at b) preinjection, c) 1‐week, and d) 8‐week postinjection (Movie S6, Supporting Information). Changes in UFD vascular parameters over time: e) vessel volume occupancy (VVO), f) fractional moving blood volume (FMBV), g) vessel number density (VND), and h) vessel tortuosity (VT). In vitro validation shows i) sustained high blood glucose (BG) and j) reduced estimated glomerular filtration rate (eGFR), calculated from serum creatinine (sCr), blood urea nitrogen (BUN), and body weight. k) Quantitative histopathology results given as normal glomeruli ratio and microvessel density remarks renal microcirculatory disorder from DKD group (Figure S9, Supporting Information). Data are plotted as mean ± standard errors.

Next, streptozotocin (STZ)‐induced diabetic rat models were followed up for 8 weeks to monitor long‐term changes in the renal microcirculation associated with DKD progression. The experimental protocol is represented in **Figure** **4**a. To induce type‐I diabetes, STZ‐citrate buffer solution was administered to four SD rats via intraperitoneal injection, and an equal volume of saline was similarly administered to a control group (*n* = 4). STZ selectively destroys β islets of the pancreas, which manifests as hyperglycemia and hypoinsulinemia.^[^ ^37^ ^]^ The rats’ kidneys were imaged at preinjection and in the 1st, 3rd, 5th, 7th, and 8th weeks after injection. All subjects were monitored for the identical blood serum biomarkers used for the AKI model, and we additionally measured the blood glucose (BG) level to ensure that hyperglycemia was sustained throughout the entire experimental period. The rats were euthanized after the 8‐week imaging, and their collected kidneys were processed for histological examination.

Compared to the densely sprouting ILAVs in the preinjection state (Figure 4b), a moderate regression of ILAVs is seen one week after the STZ injection (Figure 4c), but it is less severe than in the AKI cases (Figure 3c,d). The cause might be a temporary vascular impairment caused by the mild nephrotoxicity of STZ.^[^ ^37^ ^]^ The reduced vascular density appears to be gradually restored, but the restoration remains incomplete and the UFD volume in the 8th week appears sparser than in the preinjection state (Figure 4d; Figure S6 and Movie S6, Supporting Information). The changes in the UFD parameters over time—incomplete vascular recovery and increasing vascular complexity—indicate vascular degeneration in the diabetic group (Figure 4e–h). VVO (Figure 4e), FMBV (Figure 4f), and VND (Figure 4g) all decrease, and VT increases (Figure 4h). The biggest decreases from the preinjection state are in VVO (−16%) and FMBV (−17%) in the 1st week, and VND (−21%) in the 3rd week. After that, VVO, FMBV, and VND slowly but continuously increased, recovering to ≈89% of the preinjection state by week 8. In contrast, VVO, FMBV, and VND in the normal group slowly but steadily increase until week 8, attaining an ≈7% increase from the preinjection state. Inferred from the body weight increase in the normal group, these trends are attributed to vascular enrichment by kidney growth during the experimental period, whereas the body weights in the diabetic group either did not change much or decreased (Figure S7, Supporting Information). Finally, the UFD parameters of the diabetic kidneys in the 8th week reached ≈77% of their values for the normal kidneys at the same time point. Different from the other parameters, in the 7th week, the VT of the diabetic group had increased by 9% over the preinjection value (Figure 4h). On the other hand, in the normal group, the VT tended to decrease. The logic underlying the change of VT in the AKI model also applies here: the growing proportion of straight ILAVs reduces the average VT. In summary, the changes in UFD parameters under prolonged diabetes signify decreased renal circulation and vascular structural deformation into tortuous shapes. The results agree with the confirmed microvascular decline observed in an *ex vivo* angiographic study.^[^ ^8^ ^]^ They also reflect the reduced RBF caused by impaired autoregulation in STZ‐induced diabetic rats without insulin treatment.^[^ ^6^ ^]^

In vitro blood tests of the diabetic group show elevated BG levels in the 1st week and continuing high levels (>400 mg dL^−1^) throughout the rest of the period (Figure 4i). Along with the sustained hyperglycemia, the increases in sCr and BUN throughout the 8 weeks indicate the progression of DKD complications (Figure S8, Supporting Information). The eGFR shows the renal function decline in the diabetic group, in contrast to the increasing trend in the normal group (Figure 4j). In the 8th week, the filtration rate of the diabetic group (2.34 ± 0.28 mL min^−1^) is only 48% of that of the normal group (4.85 ± 0.35 mL min^−1^). From the histopathology, the normal glomeruli ratio and microvessel density of the DKD group are decreased by 62.6% and 60.5% than those of the normal group (Figure 4k). In the detailed look, histopathology reveals characteristic diabetic lesions in the DKD sections, which include diffuse thickening of the glomerular basement membrane and mesangial expansion within glomeruli, along with pronounced fibrotic impairment in blood vessels, as compared to normal kidneys (Figure S9, Supporting Information). In conclusion, the malignant changes in UFD parameters are attributed to kidney dysfunction resulting from diabetic complications, as validated by in vitro tests and histopathology.

## 3 Discussion

This study reports the preclinical results of contrast agent‐free ultrafast 3D imaging of renal vasculature in vivo and finds distinctive pathological vascular changes in the acute and chronic forms of kidney damage. Benefiting from high vascular flow sensitivity without any contrast agent, 3D UFD imaging can visualize the full renal vessel hierarchy, down to fine vessels as small as 167 µm. Four UFD parameters —VVO, FMBV, VND, and VT— are used to quantify the hemodynamic and morphological features in renally diseased rats. We found different parametric changes in rhabdomyolytic AKI and type‐I DKD: the hemodynamic changes are rapid and severe in the former case, while the latter exhibits chronic changes in vascular structures.

The trend of the UFD parameters in the AKI group is characterized by a rapid decline in renal circulation, associated with rapid kidney destruction. These parameter value changes agree well with in vitro serum test results, confirming a severe loss of filtration function. Along with tubular lesions observed via histopathology, major decreases in VVO, FMBV, and VND indicate blood flow reduction induced by tubular necrosis. These are well‐known manifestations of rhabdomyolysis‐related damage. We speculate that a temporary increase in VT may contribute to transient glomerular and tubular stenosis due to excessive myoglobin, particularly in the renal cortex, where ILAVs are densely populated.

Although rapid vascular rarefaction was identified in AKI rats, less prominent vascular decline was observed in the diabetic group. There was a vascular decline in the early phase, which we considered to be a transient response to STZ nephrotoxicity, but more extended observation lasting until week 8 showed mild but clear decreases in VVO, FMBV, and VND, along with a constant increase in VT. These trends describe a potential chronic vascular degradation, characterized by declining hemodynamics and vascular deformation into tortuous structures. Such findings are consistent with microvascular decline, as validated by an *ex vivo* angiographic study.^[^ ^8^ ^]^ The VT increase in the kidney is also considered as a representative diabetic manifestation, because a similar phenomenon has been observed in diabetic retinopathy.^[^ ^38^, ^39^ ^]^ Together with a reduction in eGFR, the reduced hemodynamics can be explained by the functional deterioration of the kidney. Interestingly, the recovery trend in two hemodynamic UFD parameters (VVO and FMBV) correlates well with that of eGFR. This observation may imply glomerular hyperfiltration, a primary factor in DKD pathogenesis that induces irreversible glomerular damage.^[^ ^40^, ^41^, ^42^ ^]^ Based on histopathological observations of glomerular lesions, the kidney was confirmed as DKD, showing that UFD parameters can effectively detect the microcirculatory alterations occurring in DKD progression. During the 8‐week long observation period, we observed a mild but consistent decrease in the absolute PD intensity in the normal group, attributed to increased acoustic attenuation caused by skin layer thickening by growth. To provide a measure of perfusion levels that is independent of growth changes, we applied the FMBV, which is a relative measure normalized by strong blood signals from major arteries and veins. The correlation between the FMBV and vascular flow rate is adequately verified in previous studies.^[^ ^43^, ^44^ ^]^ The FMBV in the normal group gradually increases over the period of 8 weeks, effectively reflecting a renal vascular flow that increases with the normal growth of rats.

The purpose of our study was to long‐term (max. 8‐week) follow‐up pathologic vascular change from the same subject without the use of a contrast agent, therefore, we had to carefully determine our cross‐validative modality with minimized side effects over our induced nephropathy. Contrast‐enhanced ultrasound (CEUS) involves injection of an exogenous microbubble, nevertheless, fine blood vessels are inobservable due to their lacking spatial resolution. Moreover, there exists a contraindication of CEUS toward a rat with nephropathy, as the inertial cavitation of microbubble may induce capillary rupture and result in permanent loss of renal function due to tubular necrosis.^[^ ^45^, ^46^, ^47^ ^]^ Contrast‐enhanced CT and MRI also lack the spatial resolution to visualize microvessels,^[^ ^48^ ^]^ and the contraindication of iodinated‐ or gadolinium‐based contrast agents in patients with renal impairment are still controversial.^[^ ^49^, ^50^, ^51^ ^]^ While contrast agent‐free MRI techniques like arterial spin labeling or 4D flow MRI may offer similar advantages to our 3D UFD, their intrinsic limitations in spatiotemporal resolution hinder their ability to accurately depict microscale vasculature—a capability demonstrated in our work.^[^ ^52^, ^53^, ^54^, ^55^ ^]^ Disregarding the inapplicability of the aforementioned techniques, our study's findings align with several preceding CEUS, MRI, and super‐resolution USI studies with the same or similar AKI and DKD preclinical models.^[^ ^32^, ^56^, ^57^, ^58^ ^]^ Instead, we exploited eGFR, a clinically‐prevalent parameter, as a cross‐validative measure of kidney perfusion. The correlation between eGFR and nephropathy under AKI and DKD is well established in clinics.^[^ ^59^, ^60^, ^61^ ^]^ A two‐sided Student's *t*‐test result shows a significant eGFR difference between diseased and normal kidneys (Table S1 and S2, Supporting Information). Furthermore, we also performed histopathology for a definitive end‐stage validation, where we could quantitatively confirm perfusion‐related microscopic lesions from our diseased models.

In conclusion, our findings provide valuable insights into pathological changes in renal circulation. Our imaging resolution precluded the direct visualization of fine vascular hierarchy at the glomerular level, less than 50 µm. Better resolution can be possibly achieved through super‐resolution localization techniques, although this may come at the cost of increased acquisition time and require the use of contrast agents.^[^ ^56^, ^62^ ^]^ With improved resolution, we might be able to directly observe glomerular and tubular pathogenesis. Moreover, the current linear scanning method results in anisotropic resolutions in the cross‐sectional and elevational directions. A previous study attempted to achieve isotropic spatial resolution multi‐directional mechanical scanning,^[^ ^63^ ^]^ but this method required repeated scanning to obtain the UFD volume of an entire kidney. Volumetric imaging using a 2D matrix probe might reduce scanning time and achieve isotropic spatial resolution. For instance, a recent study used a row‐column array probe to successfully image the vessels in a rat brain.^[^ ^64^ ^]^ With appropriate computational power, it may be possible to accelerate the imaging time for whole kidney imaging with a feasible resolution of ≈200 µm. Furthermore, fusing UFD with additional imaging techniques may promote comprehensive observation of renal metabolic change under pathological circumstances. For instance, multispectral PAI performed in conjunction with UFD may provide additional photochemical parameters, such as hemoglobin concentration or oxygen saturation.^[^ ^65^, ^66^, ^67^, ^68^ ^]^

To expand 3D renal UFD to human clinical research, the following factors should be considered. First, using a low‐frequency probe and increasing the acoustic power by lengthening the transmit pulse length would help to extend the imaging depth without compromising image resolution. Pulse compression could be a good technique to achieve both deep imaging and fine spatial resolution.^[^ ^69^, ^70^ ^]^ Second, the acquisition time can be accelerated by employing a 2D matrix probe, or synchronization to subsidiary vital signs such as pulse rate or respiration rate. Because renal 3D UFD can provide rich vascular flow information at an unprecedented level, it can greatly enrich our understanding of circulatory changes in acute or chronic pathogenesis, or even in monitoring allograft perfusion after renal transplant surgery.^[^ ^71^, ^72^ ^]^

## 4 Experimental Section

### 3D Ultrafast US Image Acquisition and Reconstruction

In vivo kidney images of anesthetized rats were captured with a Vantage 256 high‐frequency US imaging system (Verasonics, WA, USA). The 3D US volume was acquired by sequentially translating a high‐frequency linear US array probe (L22‐14vX, Vermon, France; center frequency, 18.0 MHz; bandwidth, 44%). A transmit US pulse was parametrically designed to generate a single bipolar wavelet with a transmit frequency of 15.625 MHz. At each slice depth, 1000 frames were acquired in planewave compounding mode with seven symmetrical steering angles (0°, ±2°, ±4°, and ±6°) at a 20 000 Hz pulse repetition frequency. After the dense seven transmit and receive events, there was a resting period of 650 µs to stabilize the probe, which then completed the 1000 Hz frame rate acquisition. For 3D scanning, the probe was fixed to a motorized linear stage system controlled by a dedicated graphical user interface (GUI) (LABVIEW 2018, National Instruments, TX, USA). After the acquisition of one slice, the US system triggered a connected DAQ board (PCIe‐6321, National Instruments) to shift the probe 0.2 mm in the elevational direction. Meanwhile, the acquired 1000 US RF frames were reconstructed into 1000 IQ datasets with the Vantage reconstruction algorithm (Verasonics). To include the full boundary of the kidney, trapezoidal FOV was defined (top width, 12.8 mm; bottom width, 15.7 mm; and depth, 15.0 mm) that provided wider lateral coverage than the probe's footprint. Including the instantaneous IQ data saving, the whole US acquisition and the image reconstruction were finished in ≈7 s per elevational slice. This sequence was repeated from the cranial to the caudal ends of the kidney, taking ≈14 min to scan the entire kidney over a typical range of 24 mm.

### Automatic Ensemble Selection and US Doppler Image Processing

After scanning was completed, ensemble selection and subsequent Doppler image processing were conducted. First, out of 1000 IQ frames from each slice, the optimal 100‐frame sequences were automatically selected as an ensemble (Figure S1, Supporting Information). Motions from respiration and heartbeat were trackable from sequential mean‐square errors of neighboring B‐mode frames. Typically, a one‐second interframe error sequence contained either no or one breath, along with 4–5 heartbeats. To exclude a random respiratory motion, a two‐sided range from the maximal point (max. 500 ms) was excluded from the ensemble candidates. Then, to capture the cardiac diastole accurately, a robust criterion was employed wherein a 100‐frame sequence with the lowest interframe error difference was selected as an ensemble. Second, an SVD‐based spatiotemporal clutter filter filtered out tissue signals from the selected ensemble (Figure S2, Supporting Information). Upper and lower rank thresholds were uniformly set as 10 and 60, respectively, to retain the highest blood signal intensity and the least background noise. Finally, the power of the blood signals was averaged and logarithmically compressed to produce cross‐sectional dB‐scale PD images.

### Preparation of Diseased Rat Models

All experimental animal procedures were performed in accordance with the protocol approved by the Institutional Animal Care and Use Committee of Pohang University of Science and Technology (POSTECH‐2021‐0052‐C2, approved on 17 Feb. 2022).

*Glycerol‐induced rhabdomyolysis AKI model*: Eight 10 weeks old male SD rats with mean body weights of 341.3 ± 5.9 g, ±standard error, were randomly divided into AKI (*n* = 4) and control groups (*n* = 4). The rats were housed under standard laboratory conditions and fed standard rodent chow. Before preinjection imaging, the rats were dehydrated and fasted for 24 h. To induce rhabdomyolysis, after imaging, the experimental group received an intramuscular injection of 50 v/v% glycerol (G7757, Sigma–Aldrich, MO, USA) and saline solution at a volume of 10 mL kg^−1^ of the body weight. The dose was equally divided into two portions and injected into the femoral muscles of both hind limbs. The control group received an equal volume of pure saline. Symptoms including oliguria, anuria, or tea‐colored myoglobinuria demonstrated the successful induction of rhabdomyolysis.

*STZ‐induced type‐1 diabetes model*: Eight 8 weeks old male SD rats with mean body weights of 275.9 ± 15.3 g, ±standard error), were randomly divided into diabetic (*n* = 4) and control groups (*n* = 4), and the rats were housed under standard laboratory conditions and fed a standard rodent chow. All rats were fasted 12 h before preinjection imaging. To induce type‐I diabetes, the diabetic group received an intraperitoneal injection of 20 mg mL^−1^ of STZ in 0.09 m citrate buffer solution at a volume of 1 mL kg^−1^ of the body weight (S0130; C2488, Sigma–Aldrich). The control group received the same volume of pure citrate buffer. The diabetic group was not treated with insulin throughout the experimental period.

### Common Pre‐Imaging Protocol

Approximately 12 h before imaging, the rats were fasted, but they had free access to water. Awake rats were then initially anesthetized using a chamber connected to a preclinical anesthetic gas system (VIP 3000 Veterinary Vaporizer, Midmark, OH, USA) supplying a 3–5% isoflurane/oxygen concentration, at a 1.5 L min^−1^ flow rate. Throughout the experiment, the anesthesia was sustained using a lab‐made nasal mask, and the isoflurane concentration was continuously regulated between 1.0% and 1.5% to maintain a stable breathing rate of 1 Hz. After the abdominal hair was removed using clippers and depilatory cream, 0.5–1 mL of blood was sampled (see Section 4.6), and the rat's body weight was recorded. The animal was then placed supine on an animal heating pad maintaining body temperature. The rat's position was fixed with a medical bandage (Micropore, 3 m, MN, USA), and the rib cage was lifted up to expose the kidney without skeletal shading. Imaging was performed on the right kidney in transverse view, scanning from the cranial to the caudal ends. The probe's position was adjusted using the aforementioned GUI panel to select the scanning range. At the starting point, a superior aspect of the kidney capsule surrounded by the right distal liver lobe was displayed. At the stopping point, the neighboring small intestine fills the void of the inferior aspect margin of the kidney.

### Multiparametric 3D UFD Analysis

Before quantification, the entire kidney volume was first segmented manually based on B‐mode and PD images, using MATLAB Volume Segmenter (MATLAB R2022a, Mathworks, MA, USA). To ensure fair comparisons of all subjects, all labeled PD volumes were normalized by the top 1% average intensity of the preinjection PD volume. The normalized PD volumes in the range of 18 dB were then rescaled and subjected to UFD parameter analyses. The quantitative analyses were divided into volumetric hemodynamics and vessel skeletal morphology. In the volumetric hemodynamics analysis, adaptive thresholding (MATLAB function “imbinarize”) was applied to the rescaled PD volume to obtain the binary kidney vessel mask, which helped to distinguish vessels and eliminate background noise. Following the definition previously devised by Tong et al.,^[^ ^73^ ^]^ the vessel mask volume *V*_vessel_ was divided by the kidney mask volume *V*_kidney_ to calculate VVO:

(1) $$\mathrm{VVO}\;=\;\frac{V_{\mathrm{Vessel}}}{V_{\mathrm{kidney}}}\;$$

A method originally described by Rubin et al. was employed to quantify FMBV.^[^ ^43^, ^44^ ^]^ FMBV indicates the relative perfusion level normalized by the high blood signals from major arteries and veins. Specifically, the PD volume is linearly scaled, assigning intensities above −6 dB a blood‐filled value of 100%, and those below −18 dB a value of 0%. The sum of the relative PD intensities (rPD) was divided by *V*_kidney_, yielding:

(2) $$\mathrm{FMBV}\;=\;\frac{\sum \left(\mathrm{rPD}\right)}{V_{\mathrm{kidney}}}$$

For vessel skeletal morphology analysis, the Hessian‐based Frangi vesselness‐filtered PD volume was added to the rescaled PD volume to enhance the vessel structure. The vesselness‐added PD volume was binarized into a vessel mask with adaptive thresholding, and the vessels’ centerlines were extracted (MATLAB function “bwskel”). To eliminate inaccurately extracted vessel segments, skeletons with branch lengths below 10 pixels were removed (MATLAB function 'bwmorph3’). Following the original article by Tong et al.,^[^ ^73^ ^]^ The VND value was calculated by dividing the total number of vessel branches (*N*_b_) by *V*_kidney_:

(3) $$\mathrm{VND}\;=\;\frac{N_{b}}{V_{\mathrm{kidney}}}$$

VT was quantified by applying a distance metric scheme at each vessel branch, which was previously devised by Bullitt et al.^[^ ^74^ ^]^ For each branch, the ratio of the actual vessel branch length, *L*_b_, and its linear distance between two endpoints, *d*_b_, was calculated. Finally, VT was calculated by dividing the sum of the ratios at every branch by *N*_b_:

(4) $$\mathrm{VT}\;=\;\frac{\sum (L_{b}/d_{b})}{N_{b}}$$

### In Vitro Blood Sample Tests

Before imaging, blood samples were collected from all rats under anesthesia. Using a heparinized capillary tube (Marienfeld HSU‐29 00 000, Germany), 0.5–1 mL of blood was drained from the retro‐orbital sinus and stored at room temperature for 30 min to induce coagulation. For diabetic experiments, the BG level was measured on the spot with a glucometer (Accu‐Chek Guide Set, Roche Diabetes Care GmbH, Germany). The sampled blood was microcentrifuged for 10 min at 10 000 g and 4 °C, and the supernatant (serum) was separated and stored in a deep freezer at below −80 °C. The sCr and BUN concentrations were measured from thawed blood serum samples using an automatic biochemical analyzer (DRI‐CHEM NX700, Fujifilm, Japan) with paired biochemical test slides (DRI‐CHEM SLIDE, Fujifilm, Japan).

### Quantitative Histopathology

After the final imaging (AKI, at 72 h; DKD, in the 8th week), the rats were sacrificed via cervical dislocation. The collected right kidneys were instantly transversely halved and stored in neutral buffered 10% formalin solution (HT501128, Sigma–Aldrich) for tissue fixation. After 2–3 weeks of preservation, the fixed kidney tissues were dehydrated and embedded in paraffin wax for histological sectioning. Three sequential transverse slices in the middle plane of the kidney were sectioned into 2–3 µm thick slices using a microtome. The sectioned kidneys underwent staining with Masson's trichrome, PAS, and CD31 immunostaining. They were examined using a light microscope (ECLIPSE Si RS, Nikon, Japan) at magnifications of ×4 or ×10, with guidance from a practiced medical doctor. First, the perfusion‐related lesions are well demonstrated from the microscopic images of Masson's trichrome‐stained kidney sections. Then, a quantitative histological analysis was followed on PAS‐ and CD31 immunostained slides, focusing on two parameters: the normal glomeruli ratio and microvessel density. Seven regions of interest (ROIs) were randomly selected from the cortical region of every PAS‐stained and CD31‐immunostained kidney slice (*n* = 4 per group). The normal glomeruli ratio was measured from the PAS slide counting the total number of glomeruli present in all 7 ROIs (≈100–150 glomeruli per slide), and the number of normal glomeruli among them. To determine the microvessel density, the average proportion of brown pixels (vascular endothelial cells) on the CD31 slides was measured using a color extraction function integrated into software (ImageJ, National Institute of Health, MD, USA). In the AKI experiment, a normal tubule ratio was additionally included as a parameter, due to the tubular necrosis resulting from rhabdomyolysis‐induced etiology. The method closely mirrored that of the normal glomeruli ratio, enrolling ≈220–330 tubular cells per slide in a ×10 PAS‐stained image.

### Statistical Analysis

The significance difference between the diseased kidneys (*n* = 4 AKI, *n* = 4 DKD) and the healthy kidneys (*n* = 4 AKI control, *n* = 4 DKD control) were assessed using a two‐tailed Student's *t*‐test (unequal variances) on all parameters including UFD (VVO, FMBV, VND, VT), in vitro (eGFR, BG) and histopathology (normal glomeruli ratio, normal tubule ratio, and microvessel density). The statistical output was calculated from the raw data and presented as mean ± standard error. To further validate the statistical significance, a two‐sided Student's *t*‐test was employed. For each *t*‐test, a null hypothesis (*H*_0_) was set as an inseparable difference between the diseased and normal kidneys. A *p*‐value lower than 0.05 was set as a moderate rejecting criterion of *H*_0_ at a 5% significance level. Additionally, *p*‐values lower than 0.01 were considered to denote greater significance. The statistical analysis was performed using the “ttest” built‐in function in Excel (Microsoft, WA, USA).

## Conflict of Interest

The authors declare no conflict of interest.

## Supporting information

Supporting Information

Supplemental Movie 1

Supplemental Movie 2

Supplemental Movie 3

Supplemental Movie 4

Supplemental Movie 5

Supplemental Movie 6

## Data Availability

The data that support the findings of this study are available from the corresponding author upon reasonable request.
