# Supplementary material for: Contrast Agent‐Free 3D Renal Ultrafast Doppler Imaging Reveals Vascular Dysfunction in Acute and Diabetic Kidney Diseases
Source: Adv Sci (Weinh). 2023 Oct 17;10(36):2303966. doi: 10.1002/advs.202303966 (PMC10754092; doi:10.1002/advs.202303966)
Supplement: Supplementary file 1 — Supporting Information [file ADVS-10-2303966-s006.pdf]

ADVANCED SCIENCE
Open Access

Supporting Information

for *Adv. Sci.*, DOI 10.1002/advs.202303966

Contrast Agent-Free 3D Renal Ultrafast Doppler Imaging Reveals Vascular Dysfunction in Acute and Diabetic Kidney Diseases

Donghyeon Oh, Donghyun Lee, Jinseok Heo, Jooyoung Kweon, Uijung Yong, Jinah Jang, Yong Joo Ahn* and Chulhong Kim**

Supporting Information

Contrast Agent-free 3D Renal Ultrafast Doppler Imaging Reveals Vascular Dysfunction in Acute and Diabetic Kidney Diseases

Donghyeon Oh, Donghyun Lee, Jinseok Heo, Jooyoung Kweon, Uijung Yong, Jinah Jang, Yong Joo Ahn*, and Chulhong Kim**

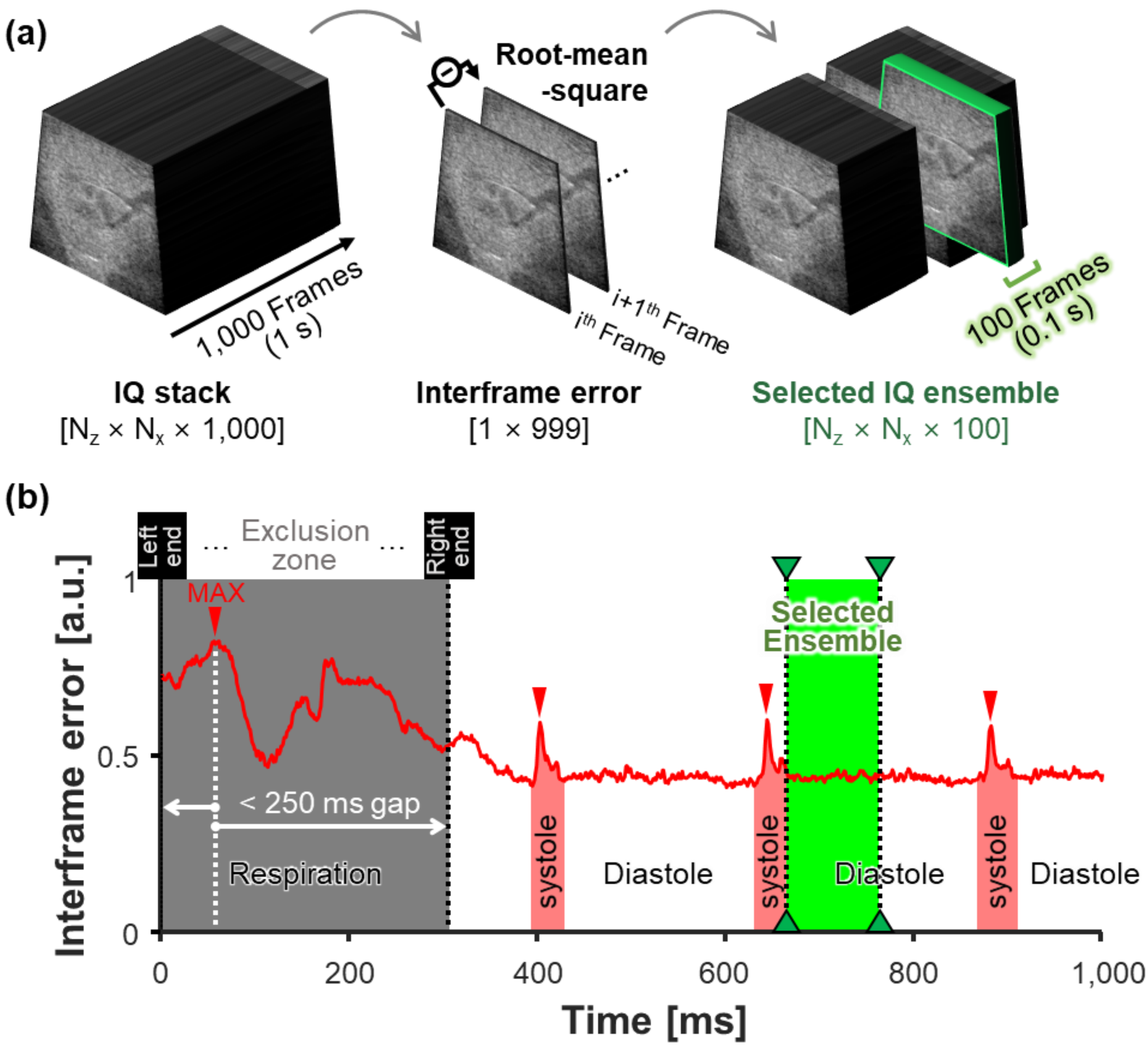

Figure S1. Interframe error-based automatic ensemble selection algorithm. (a) A schematic describing interframe error calculation from 1,000-frame IQ stack. (b) Windowing criteria for the ensemble selection. Interframe error describes the respiratory and cardiac motion during imaging time. A two-sided range (max. 500 ms) from the maximal point is excluded, regarding as a respiratory motion. Among the remaining sequential frames, a section with the least interframe error difference is selected as an ensemble. IQ, in-phase quadrature data; N_z, axial FOV pixel length; N_x, lateral axial FOV pixel length; a.u., arbitrary unit.

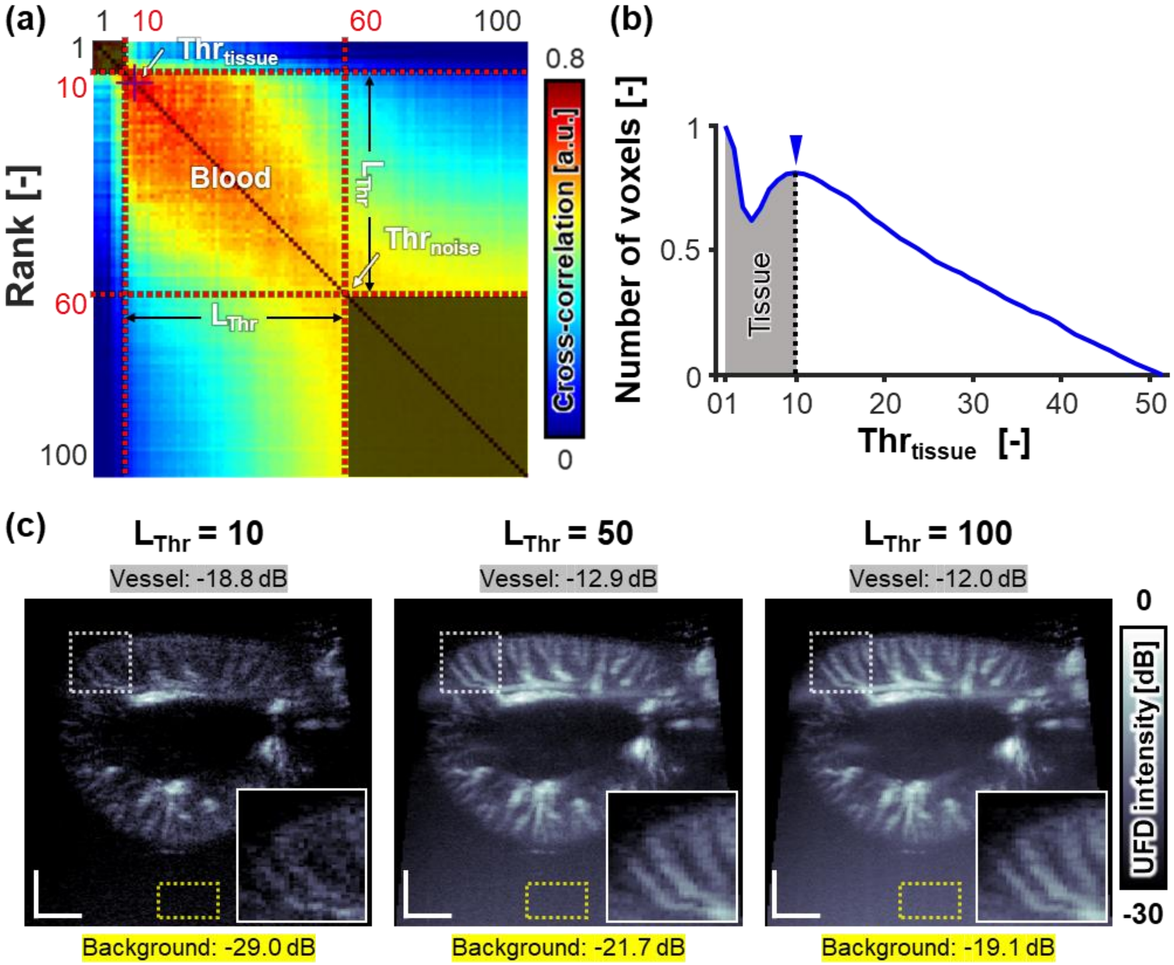

Figure S2. Dual rank thresholding criteria in SVD-based spatiotemporal clutter filtering. (a) A spatial cross-correlation matrix describing the tissue, blood, and random noise signal arranged by rank. (b) Tissue threshold (Thr_{tissue}) selection criterion from whole 3D renal PD volume. The number of voxels above -20 dB was the highest at 10 after rejection of tissue. (c) Threshold gap (L_{Thr}) selection criterion compromising between vessel contrast and background noise. Following to an increasing L_{Thr}, both the vascular contrast and the background noise level at deep region increases. Regarding both, L_{Thr} was selected as 50.

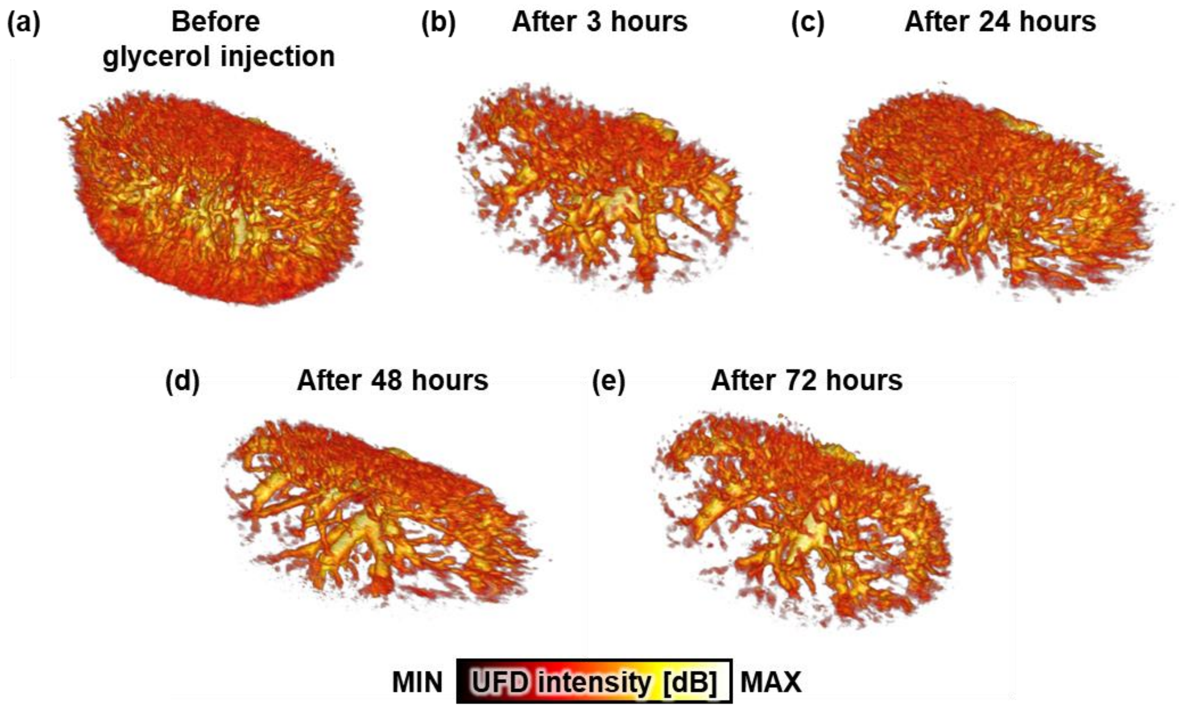

Figure S3. Progression of renal vascular rarefaction observed in the 3D renal UFD volume of a glycerol-induced AKI model. (a) Preinjection, (b) 3 hours, (c) 24 hours, (d) 48 hours and (e) 72 hours after glycerol injection.

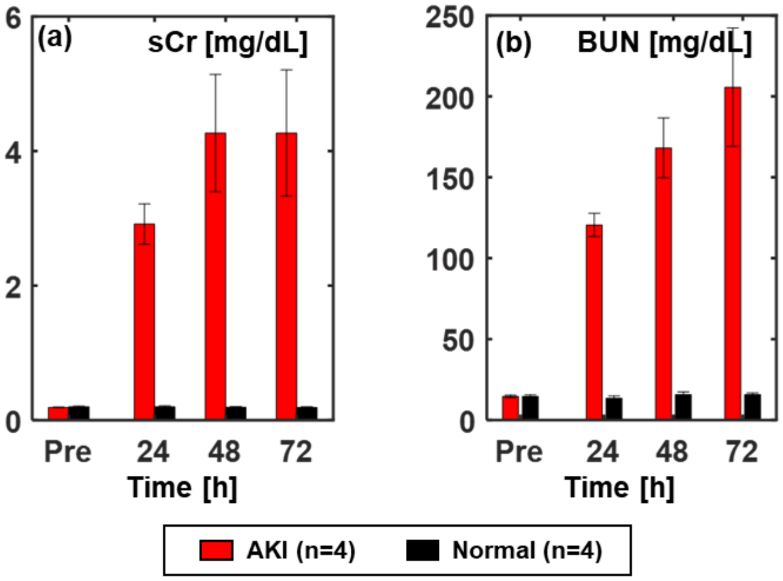

Figure S4. Validation of glycerol-induced AKI via *in vitro* blood serum tests. Increases in (a) serum creatinine concentration (sCr) and (b) blood urea nitrogen (BUN) in blood serum show the renal dysfuction over 72-hour postinjection. Error bar = standard error.

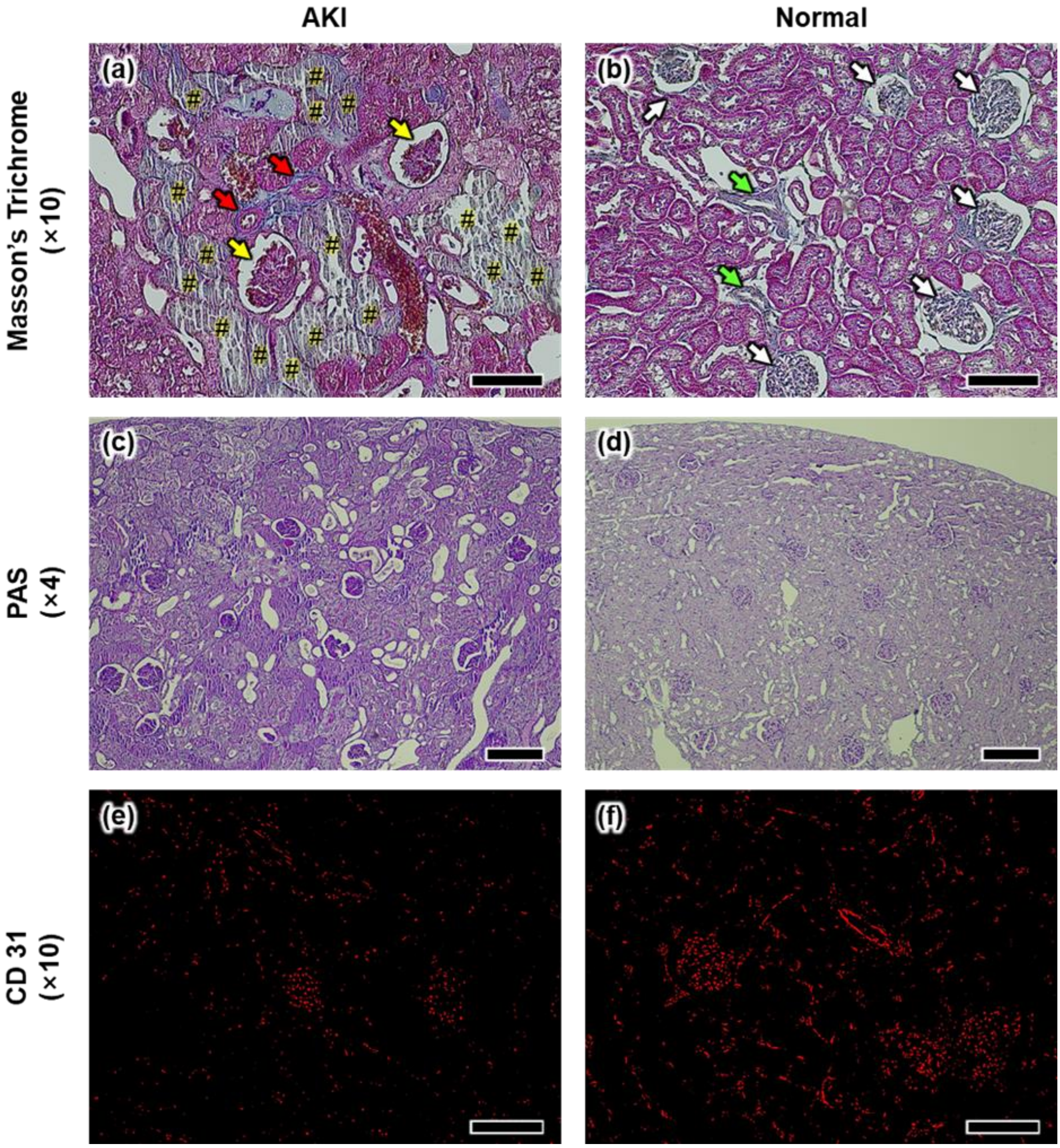

Figure S5. Histopathological microscopic images of AKI and normal kidneys. (a) AKI describes severe fibrosis from glomeruli (yellow arrow) and blood vessels (red arrow), together with major tubular necrosis (marked with #) from Masson's trichrome-stained section (×10). On the contrary, (b) a normal kidney exhibits healthy glomeruli (white arrow) with clearly margined podocytes and urinary space, and healthy blood vessels (green arrow). Scale bar = 50 μm. Global glomerular and tubular cell damages are well demonstrated from the PAS-stained section (×4) of an (c) AKI compared to that of a (d) normal kidney. Scale bar = 100 μm. From CD31 immuno-stained section (×10), endothelial cells (brown hue) are less appearing from (e) AKI section compared to (f) normal section. Scale bar = 50 μm.

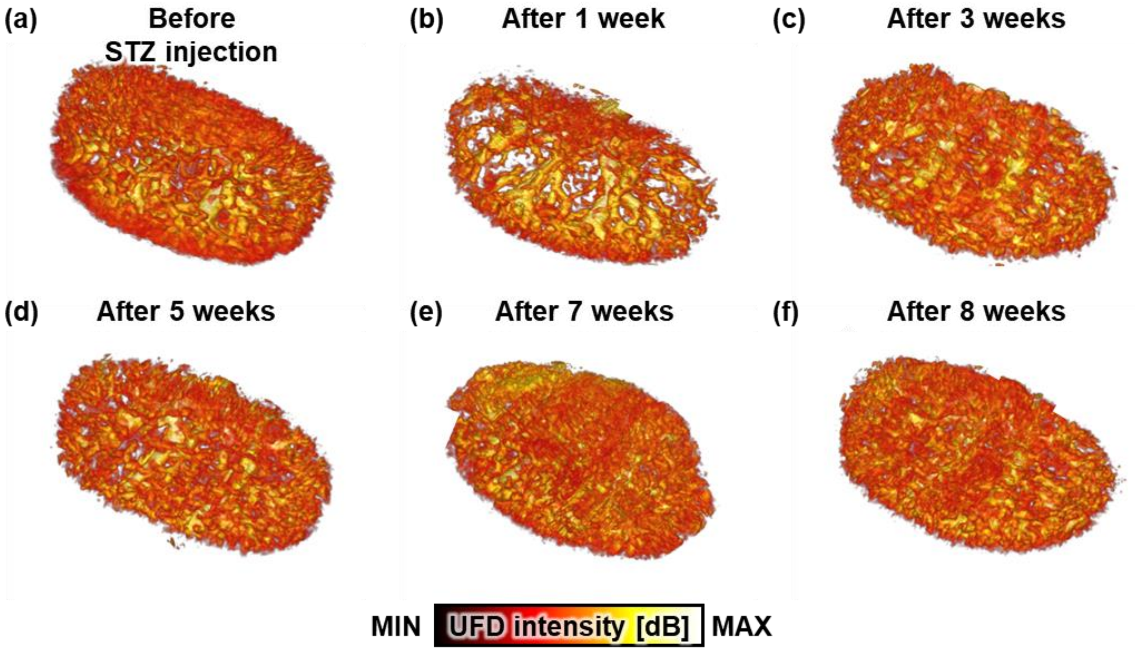

Figure S6. Full 8-week progression of renal vascular decline observed from the 3D renal UFD volume of a STZ-induced diabetic model. (a) Preinjectory state, (b) 1 week, (c) 3 weeks, (d) 5 weeks, (e) 7 weeks and (f) 8 weeks after STZ injection.

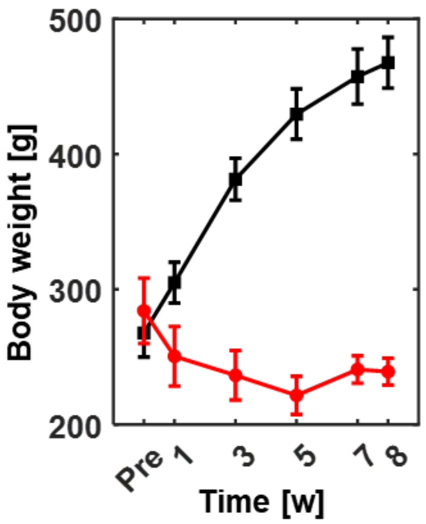

Figure S7. Body weight change in normal and diabetic rats. During an 8-week period, the control group (black) shows a nearly linear body weight increase, while the diabetic group (red) gradually loses weight after injection. Error bar = standard error.

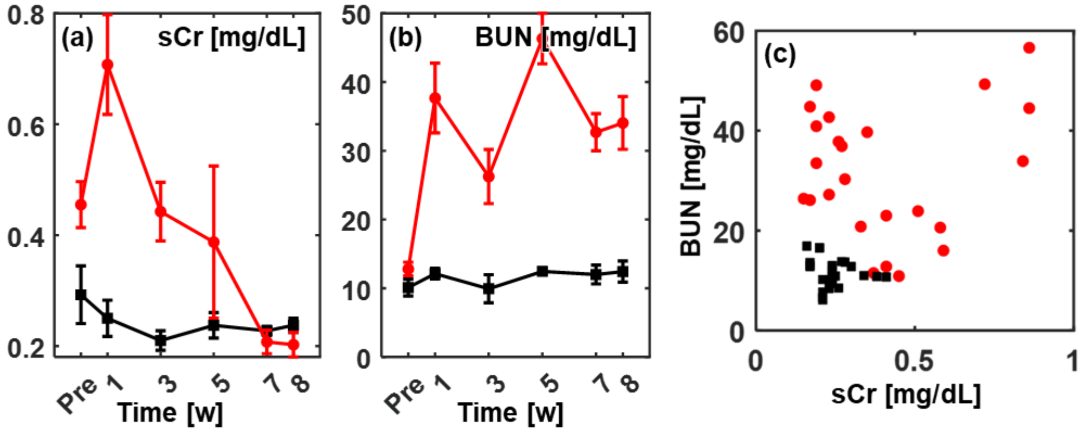

Figure S8. sCr and BUN increase as STZ-induced DKD progresses. *In vitro* serum test results for (a) the sCr concentrations and (b) BUN of a diabetic group (red) and a normal group (black). (c) sCr-BUN distributions of all blood serum results show the increases in both BUN and sCr in the diabetic group (red) compared to the normal group (black). Error bar = standard error.

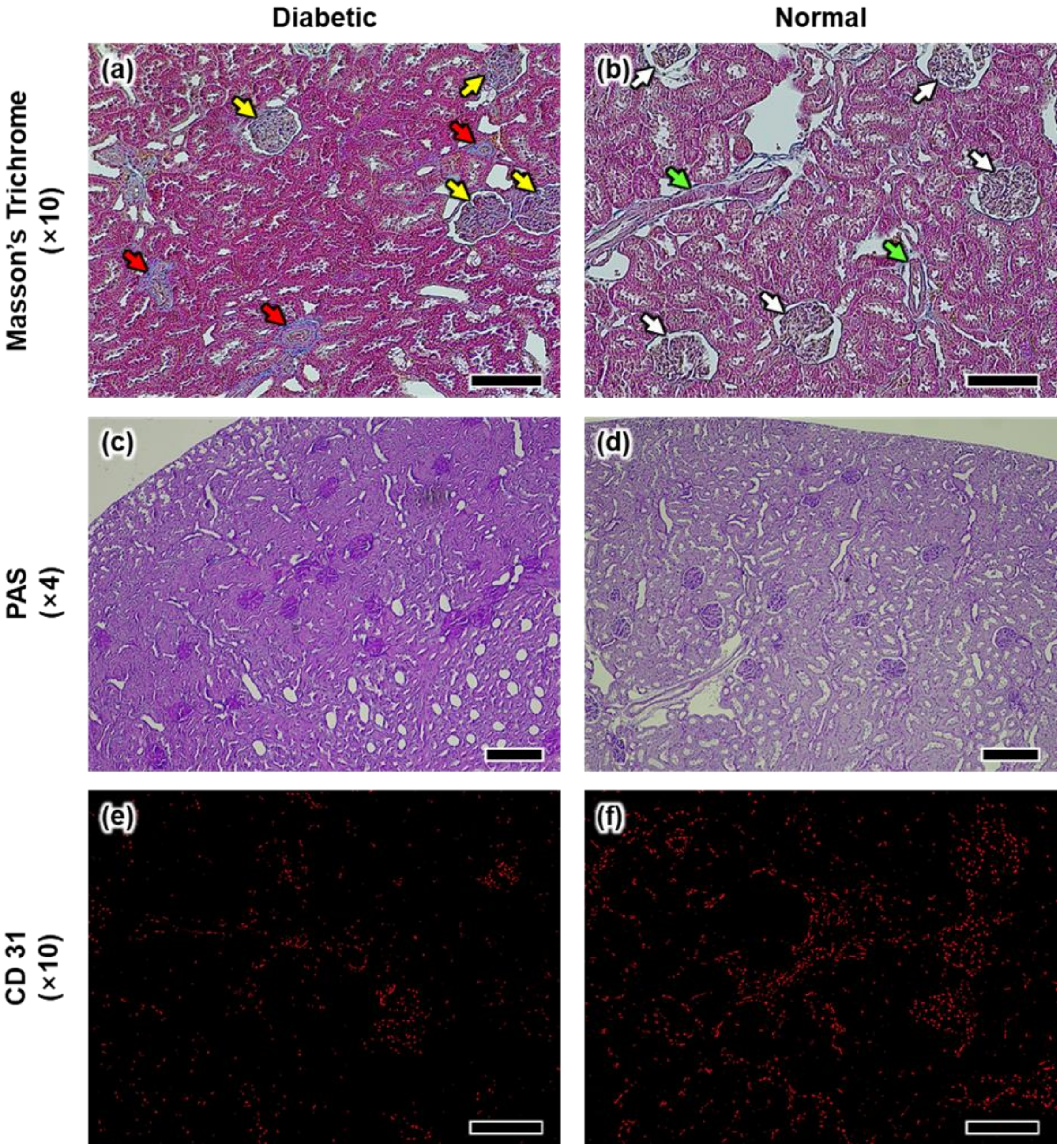

Figure S9. Histopathological microscopic images of DKD and normal kidneys. (a) DKD describes diffused glomerular basement membrane thickening and mesangial expansion in glomeruli (yellow arrow) and heavy fibrotic change in blood vessels (red arrow) from Masson's trichrome-stained section (×10). (b) A comparative normal kidney shows healthy glomeruli (white arrow) and blood vessels (green arrow). Scale bar = 50 μm. (c) A global proliferation of glomerulosclerosis and tubular atrophy from PAS-stained section (×4) characterizes DKD. (d) A comparative PAS-stained section (×4) of a normal kidney. Scale bar = 100 μm. From CD31 immuno-stained section (×10), endothelial cells (brown hue) are less appearing from (e) DKD compared to (f) normal kidney. Scale bar = 50 μm.

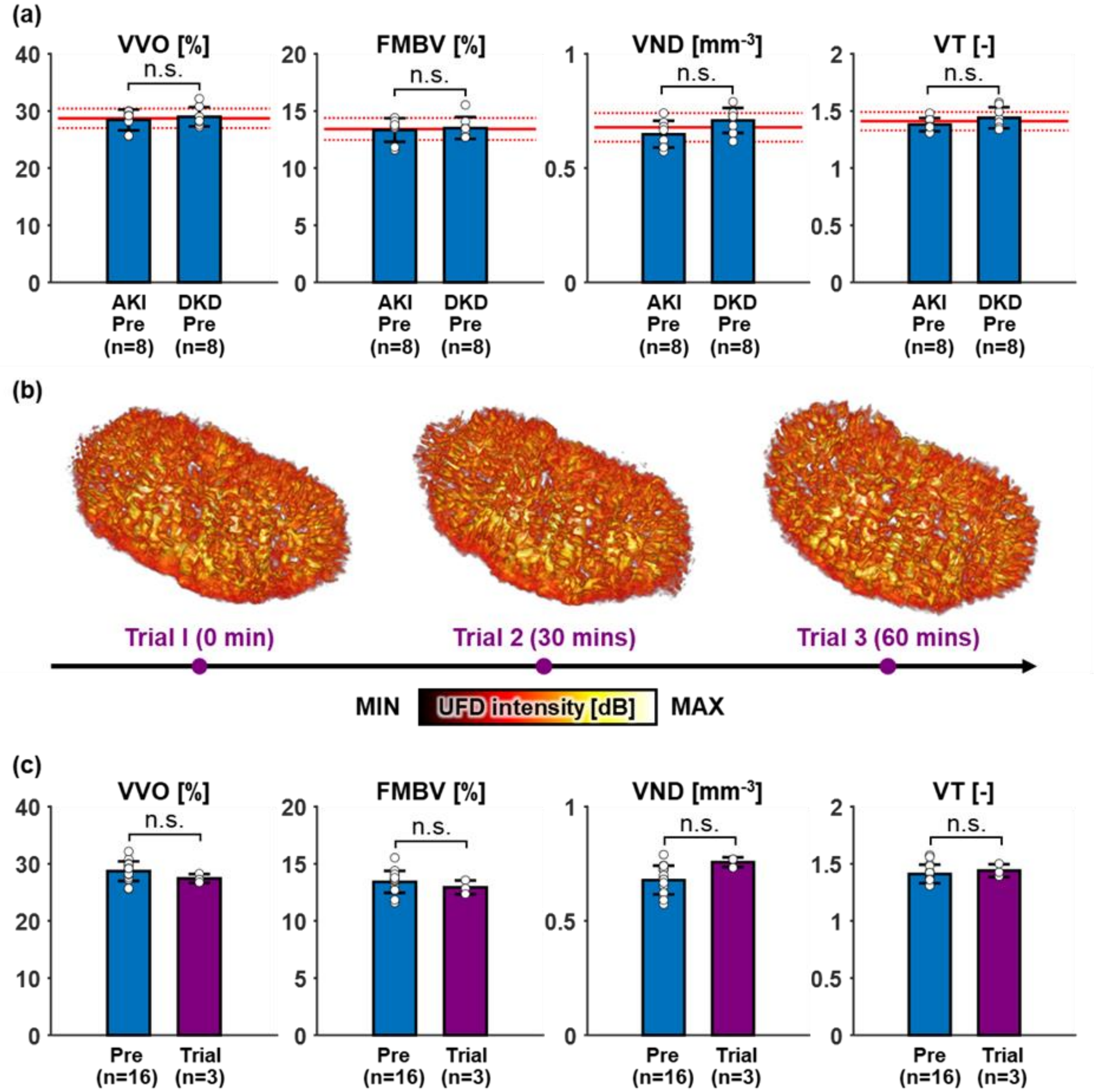

Figure S10. Inter and intrasubject repeatability examination of UFD vascular parameters. (a) Intersubject repeatability was assessed from all subjects involved in the AKI (n=8) and DKD (n=8) experiments at the preinjection state. *p*-value quantified from the student's t-test validates the inseparable (n.s.; *p*>0.05) difference between two groups. White dots represent individual data cursors, and the red bold line and dotted line represent total mean and total standard deviation, respectively (n=16). (b) Intrasubject repeatability examined from triplicated 3D UFD acquisition over the same rat in the 30-minutes time interval. (c) Comparison of UFD parameters between the repeated trial (n=3) and the preinjection state of experimental subjects (n=16). *p*-value quantified from the Student's t-test validates the inseparable difference between two groups. Error bar; standard deviation.

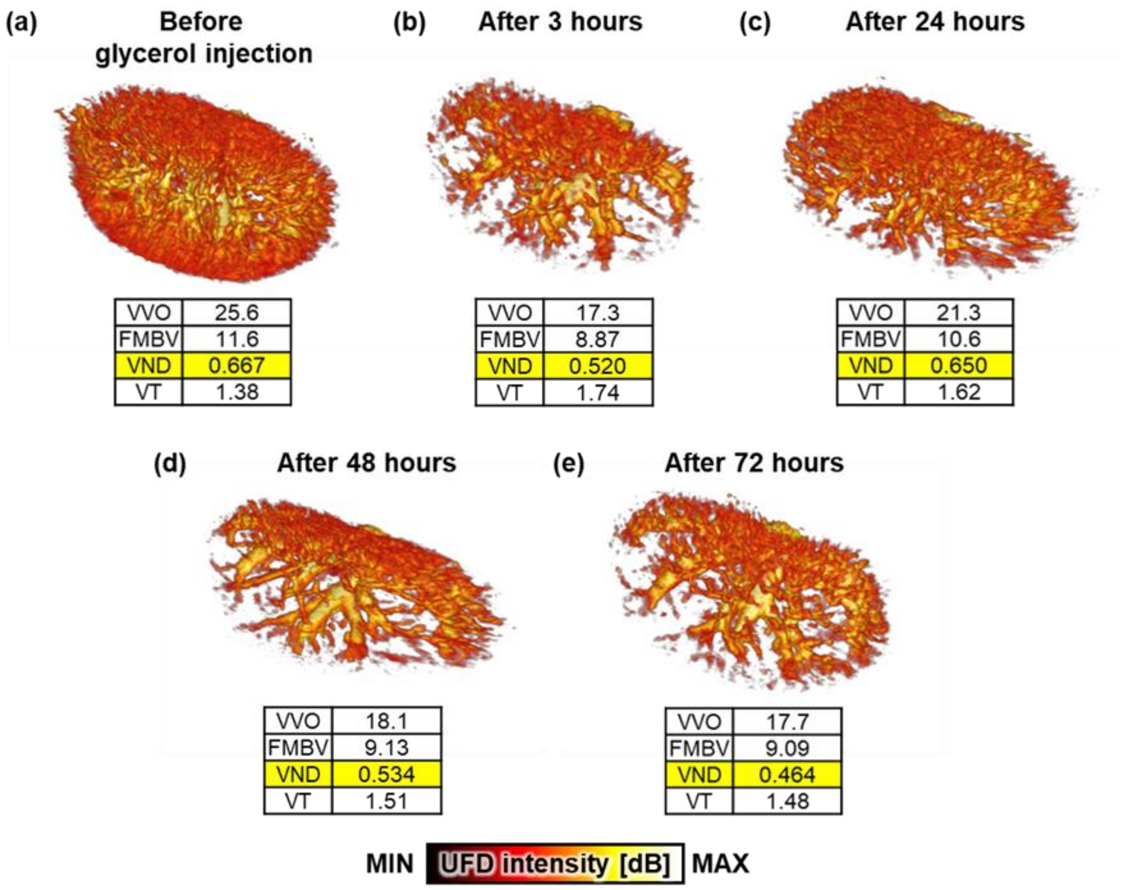

Figure S11. UFD volumes and corresponding UFD parameters of a representative AKI subject. (a) Preinjection, (b) 3 hours, (c) 24 hours, (d) 48 hours and (e) 72 hours after glycerol injection.

Table S1. Statistical analysis of UFD, in vitro, and histopathology in acute and normal group.

Parameter		Group	Duration				
			Pre	3 h	24 h	48 h	72 h
Ultrafast Doppler	VVO [%]	Acute (n=4)	28.8 ± 0.9	16.4 ± 1.8	19.7 ± 1.0	18.2 ± 0.8	17.7 ± 1.6
		Normal (n=4)	28.1 ± 0.7	29.2 ± 0.7	29.7 ± 0.4	30.0 ± 1.3	31.3 ± 0.2
		p value	0.622 (n.s.)	0.005**	0.002**	0.001**	0.004**
	FMBV [%]	Acute (n=4)	13.3 ± 0.5	9.0 ± 0.6	9.5 ± 0.6	8.7 ± 0.3	8.6 ± 0.8
		Normal (n=4)	13.3 ± 0.4	14.1 ± 0.5	14.3 ± 0.2	14.6 ± 0.9	15.5 ± 0.1
		p value	0.992 (n.s.)	0.002**	0.003**	0.008**	0.005**
	VND [mm^{-3}]	Acute (n=4)	0.698 ± 0.014	0.608 ± 0.043	0.553 ± 0.047	0.488 ± 0.017	0.481 ± 0.046
		Normal (n=4)	0.599 ± 0.010	0.684 ± 0.029	0.719 ± 0.021	0.736 ± 0.056	0.761 ± 0.017
		p value	0.003**	0.256 (n.s.)	0.048*	0.027*	0.009**
	VT [-]	Acute (n=4)	1.40 ± 0.03	1.82 ± 0.06	1.69 ± 0.07	1.50 ± 0.04	1.51 ± 0.02
		Normal (n=4)	1.36 ± 0.02	1.39 ± 0.03	1.43 ± 0.05	1.36 ± 0.02	1.43 ± 0.05
		p value	0.479 (n.s.)	0.005**	0.040*	0.058 (n.s.)	0.268 (n.s.)
In vitro	eGFR [$mL\ min^{-1}$]	Acute (n=4)	4.25± 0.14	N/A	0.15 ± 0.02	0.98 ± 0.03	0.10 ± 0.03
		Normal (n=4)	3.96 ± 0.22	N/A	4.38 ± 0.38	4.04 ± 0.31	4.00 ± 0.22
		p value	0.371 (n.s.)	-	0.002**	0.001**	< 0.001**
Histopathology	Normal glomeruli ratio [%]	Acute (n=4)	N/A				39.2 ± 8.9
		Normal (n=4)					82.1 ± 5.1
		p value					0.011*
	Normal tubule ratio [%]	Acute (n=4)	N/A				31.7 ± 3.4
		Normal (n=4)					95.5 ± 1.0
		p value					< 0.001**
	Microvessel Density [%]	Acute (n=4)	N/A				1.33 ±0.08
		Normal (n=4)					3.52 ±0.17
		p value					< 0.001**

VVO, vessel volume occupancy; FMBV, fractional moving blood volume; VND, vessel number density; VT, vessel tortuosity; eGFR, estimated glomerular filtration rate; N/A, not available. Mean ± standard error; * $p < 0.05$; ** $p < 0.01$; (n.s.) $p > 0.05$

Table S2. Statistical analysis of UFD, in vitro, and histopathology in diabetic and normal group.

Parameter		Group	Duration					
			**Pre**	**Week 1**	**Week 3**	**Week 5**	**Week 7**	**Week 8**
**Ultrafast Doppler**	**VVO [%]**	Diabetic (n=4)	28.1 ± 0.4	24.0 ± 1.6	25.3 ±1.5	25.6 ± 1.2	27.5 ± 1.3	26.0 ± 0.4
		Normal (n=4)	29.8 ± 0.9	30.0 ± 0.9	29.6 ± 0.5	31.0 ± 0.8	31.5 ± 0.3	31.3 ± 0.9
		p value	0.229 (n.s.)	0.034*	0.053 (n.s.)	0.017*	0.041*	0.004**
	**FMBV [%]**	Diabetic (n=4)	13.1 ± 0.2	10.9 ± 0.9	10.9 ± 0.6	11.3 ± 0.7	12.3 ± 0.8	11.4 ± 0.3
		Normal (n=4)	13.9 ± 0.5	14.3 ± 0.7	14.4 ± 0.3	15.2 ± 0.5	15.1 ± 0.3	15.4 ± 0.7
		p value	0.208 (n.s.)	0.036*	0.005**	0.007**	0.036*	0.003**
	**VND [mm^{-3}]**	Diabetic (n=4)	0.681 ±0.024	0.577 ± 0.035	0.540 ± 0.026	0.571 ± 0.036	0.650 ± 0. 013	0.584 ± 0.041
		Normal (n=4)	0.736 ± 0.019	0.739 ± 0.054	0.775 ± 0.031	0.743 ± 0.012	0.800 ± 0.030	0.777 ± 0.034
		p value	0.165 (n.s.)	0.074 (n.s.)	0.002**	0.007**	0.007**	0.019*
	**VT [-]**	Diabetic (n=4)	1.42 ± 0.02	1.44 ± 0.04	1.48 ± 0.01	1.52 ± 0.01	1.55 ± 0.04	1.54 ± 0.03
		Normal (n=4)	1.46 ± 0.06	1.45 ± 0.04	1.48 ± 0.05	1.39 ± 0.03	1.38 ± 0.02	1.39 ± 0.04
		p value	0.639 (n.s.)	0.801 (n.s.)	0.984 (n.s.)	0.027*	0.014*	0.030*
In vitro	**BG [mg dL^{-1}]**	Diabetic (n=4)	86.8 ± 7.4	315 ± 66	538 ± 31	462 ± 58	525 ± 9	535 ± 10
		Normal (n=4)	107 ± 5	109 ± 8	117 ± 3	136 ± 9	130 ± 7	142 ± 4
		p value	0.094 (n.s.)	0.072 (n.s.)	0.001**	0.015**	< 0.001**	< 0.001**
	**eGFR [mL min^{-1}]**	Diabetic (n=4)	2.22 ± 0.03	0.99 ± 0.29	1.52 ± 0.13	1.44 ± 0.25	2.30 ± 0.22	2.34 ± 0.28
		Normal (n=4)	3.26 ± 0.32	3.53 ± 0.23	5.04 ± 0.19	4.54 ± 0.32	4.90 ± 0.13	4.85 ± 0.35
		p value	0.079 (n.s.)	< 0.001**	< 0.001**	0.001**	< 0.001**	0.003**
**Histopathology**	**Normal glomeruli ratio [%]**	Diabetic (n=4)	N/A					30.1 ± 8.1
		Normal (n=4)						80.5 ± 4.3
		p value						0.003**
	**Microvessel density [%]**	Diabetic (n=4)	N/A					1.24 ±0.09
		Normal (n=4)						3.14 ±0.08
		p value						< 0.001**

VVO, vessel volume occupancy; FMBV, fractional moving blood volume; VND, vessel number density; VT, vessel tortuosity; BG, blood glucose level; eGFR, estimated glomerular filtration rate; N/A, not available. Mean ± standard error; * $p < 0.05$; ** $p < 0.01$; (n.s.) $p > 0.05$

Movie Legends

Movie S1. US B-mode slice sequence of a whole kidney selected by interframe error-based automatic ensemble selection.

Movie S2. US B-mode and UFD slice sequence pair of a whole kidney.

Movie S3. Step-by-step 3D renal UFD processing procedure and vascular anatomy of a rat's kidney.

Movie S4. Vascular skeleton extracted from 3D renal UFD volume.

Movie S5. Progression of vascular rarefaction in glycerol-induced rhabdomyolytic AKI rat model.

Movie S6. Progression of chronic vascular deformation in an STZ-induced type-I DKD rat model.
